# Remarkable Features of Mitochondrial DNA of Acanthamoeba polyphaga Linc Ap-1, Revealed by Whole-Genome Sequencing

**DOI:** 10.1128/MRA.00430-19

**Published:** 2019-06-20

**Authors:** Andrey V. Karlyshev

**Affiliations:** a School of Life Sciences, Pharmacy, and Chemistry, SEC Faculty, Kingston University, Kingston upon Thames, United Kingdom; University of California, Riverside

## Abstract

Whole-genome sequencing of Acanthamoeba polyphaga Linc Ap-1 resulted in a draft assembly of the chromosomal DNA and a complete sequence of the mitochondrial DNA (mtDNA). Despite very high sequence similarity with the mtDNA of Acanthamoeba castellanii Neff, in contrast to *Acanthamoeba polyphaga* Linc Ap-1, the determined DNA sequence revealed a complete absence of introns.

## ANNOUNCEMENT

Both Acanthamoeba polyphaga and Acanthamoeba castellanii are versatile organisms regulating microbial communities and are also used as models for bacterial infection (1–5). Despite the similarities in their biological properties, remarkable differences between their mitochondrial genomes were revealed.

The amoeba used for this work was *A. polyphaga* Linc Ap-1 (6). The DNA was extracted using an Invitrogen PureLink genomic DNA minikit (Thermo Fisher Scientific), according to the manufacturer’s protocol. The genome sequence was generated using a NEBNext fast fragmentation and library preparation kit, an Ion Torrent 400 sequencing kit, a template OT2 400 preparation kit, and a 316 Chip version 2. This produced 3,289,881 single-end reads (62% clonal, 3% low quality) with an average size of 282 nucleotides (nt) and a total of 926 million bases. The sequencing reads were assembled *de novo* using the Torrent SPAdes plugin version 4.4.0.1, with default parameters (uniform coverage; minimum contig size, 0.5 kb), into 18,098 contigs (0.5 to 73.3 kb) with 18.66× coverage, an *N*_50_ value of 4,176 bases, and a total assembly size of 49.35 Mb with a 58.1% G+C content. For comparison, the whole genome of A. castellanii Neff is 42.02 Mb long, with 57.8% G+C content (GenBank accession number AHJI00000000). The previously reported size of the *A. polyphaga* genome (120 Mb; GenBank accession number CDFK00000000) seems to be an overestimate due to a large number of contigs (224,482) containing very short sequences, suggesting assembly issues. Construction of the mitochondrial DNA (mtDNA) genome sequence was assisted by read mapping onto the mtDNA of A. castellanii strain Neff (GenBank accession number NC_001637) (7). Considering the highly similar gene content (except for introns and hypothetical genes), as well as the identical gene synteny and very high sequence similarity between the genes in these DNAs, the gene annotation in the mtDNA of *A. polyphaga* was derived from that in the mtDNA of A. castellanii. The total size was 39,215 bp, with 2,489.68× coverage. Comparison with the whole-genome coverage suggests approximately 132 mitochondria/cell. The G+C content of *A. polyphaga* mtDNA is 29.0%, which is comparable to that of A. castellanii Neff mtDNA (29.4% G+C content) (6).

**FIG 1:**
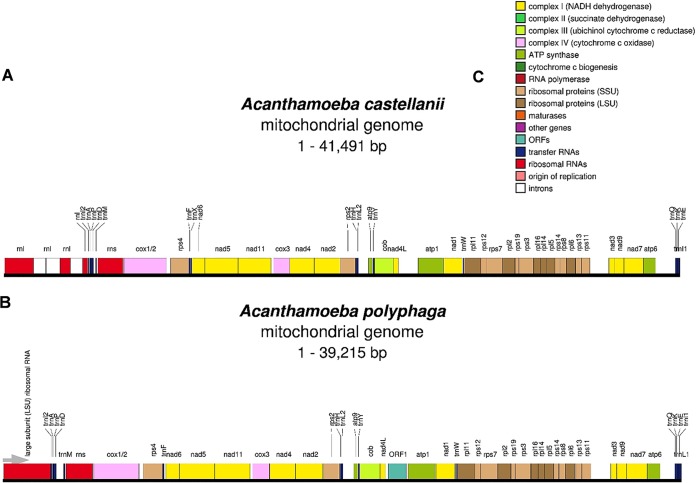
Comparison of genetic maps of mtDNA of *A. polyphaga* and A. castellanii. (A) A. castellanii mtDNA; the *rnl* gene encoding large ribosomal subunit (LSU) contains three introns (open boxes). (B) A. polyphaga mtDNA; “large subunit (LSU) ribosomal RNA” marks the *rnl* gene in red on the left. (C) Color coding for the genes according to their functions. ORFs, open reading frames; SSU, small subunit.

Currently, the only other available complete sequence of mtDNA of an *Acanthomoeba* species is that of A. castellanii (7), with the total size of 41,591 bp, which is over 2 kb larger than that of the mtDNA of *A. polyphaga*. Comparative analysis of these sequences (Fig. 1) using OrganellarGenomeDRAW (OGDRAW) software version 1.3.1 (https://chlorobox.mpimp-golm.mpg.de/OGDraw.html) (8) revealed that this difference is mainly attributed to introns present in the gene encoding the large ribosomal subunit of A. castellanii, which are not found in *A. polyphaga* DNA.

The finding suggests that these species are evolutionarily divergent, although it is difficult to say whether the difference is due to intron loss (*A. polyphaga*) or acquisition (A. castellanii). In other organisms, the introns in the *rnl* genes encoding large rRNAs are important for ribosomal assembly, and their loss may result in reduced fitness (9). Interestingly, the loss of introns is typical for mtDNA in vertebrates, with human mtDNA also containing no introns (reviewed in reference 10). Whether the lack of introns in *A. polyphaga* mtDNA affects the biological properties of this microorganism requires further investigation to determine.

### Data availability.

The whole-genome shotgun sequence of Acanthamoeba polyphaga strain Linc Ap-1 and the complete sequence of its mtDNA have been deposited in GenBank under the accession numbers LQHA00000000 and KP054475, respectively. The raw data have been deposited in GenBank under SRA number SRP191763.
